# Comparison of implementation success across three national initiatives to improve veterans' access to specialty care

**DOI:** 10.1186/1748-5908-10-S1-A9

**Published:** 2015-08-14

**Authors:** Julie C Lowery

**Affiliations:** VA Center for Clinical Management Research, VA Ann Arbor Healthcare System, Ann Arbor, MI 48189 USA

## Objectives

The Veterans Health Administration (VHA) has conducted a nation-wide implementation of three major initiatives for improving Veterans' access to specialty care in the past 2-3 years. The objective of this study was to identify the key factors affecting implementation success across these different initiatives.

## Methods

The Consolidated Framework for Implementation Research (CFIR) was used as the basis for defining potential factors affecting implementation success and guided data collection and analysis. For each of the initiatives, a sample of facilities was selected to maximize variability in implementation success, defined uniquely for each initiative. Semi-structured interviews were conducted with key stakeholders from each of the facilities. Interview responses were coded deductively based on the CFIR and were used to obtain an overall rating for each site, by construct. Site ratings were dichotomized and analyzed using crisp set Qualitative Comparative Analysis (cs-QCA).

## Findings

129 interview responses from 23 sites were coded and analyzed. CFIR constructs for which data were available consistently across all three initiatives included adaptability, compatibility, design quality and packaging, networks and communications, leadership engagement, available resources, and reflecting and evaluating. Across the three initiatives, the construct with the greatest necessity score was adaptability; 92% of sites with high implementation success rated positive for this construct. Compatibility also scored high (83%) on necessity. The constructs with the greatest sufficiency scores were leadership engagement as well as reflecting and evaluating; of all of those sites who had a positive rating for either of these constructs, two thirds achieved high implementation success. Findings varied by initiative; leadership engagement, as well as networks and communications, were more important for successful implementation of the most complex initiative (Specialty Care Neighborhood) than for the other two initiatives. Certain combinations of constructs guaranteed high implementation success.

